# Co-morbid biomarkers for sarcopenic obesity associated with gut microbiota metabolites: From burden to treatment

**DOI:** 10.1371/journal.pcbi.1014225

**Published:** 2026-05-04

**Authors:** Juehan Wang, Haijun Li, Weiyi Shi, Xiaoxu Ren, Yingying Liu, Lin Mao, Daming Wang, Tianfang Zhang, Ziwei Zhang, Huiqin Zheng, Xiaofeng Yang, Mingfei Yao, Zuobing Chen

**Affiliations:** 1 Departments of Physical Medicine and Rehabilitation, Zhejiang University School of Medicine First Affiliated Hospital, Hangzhou, China; 2 Department of Critical Care Medicine, Zhejiang University School of Medicine First Affiliated Hospital, Hangzhou, China; 3 Emergency and trauma center, Zhejiang University School of Medicine First Affiliated Hospital, Hangzhou, China; 4 State Key Laboratory for Diagnosis and Treatment of Infectious Diseases, National Clinical Research Center for Infectious Diseases, Collaborative Innovation Center for Diagnosis and Treatment of Infectious Diseases, Zhejiang University School of Medicine First Affiliated Hospital, Hangzhou, China; 5 Jinan Microecological Biomedicine Shandong Laboratory, Jinan, China; 6 Zhejiang Key Laboratory of Intelligent Rehabilitation and Translational Neuroelectronics, Hangzhou, China; Indonesia International Institute for Life Sciences, INDONESIA

## Abstract

**Background:**

The detrimental cycle of sarcopenic obesity (SO) significantly reduces quality of life in older adults, while the mechanisms are still unclear.

**Materials and methods:**

We first analyzed the incidence of SO using the CHARLS database. We identified key genes by integrating differentially expressed genes, weighted gene co-expression network analysis, and targets of gut microbiota metabolites, refining the selection through machine learning methods (LASSO, XGBoost, SVM-REF, Random Forest). These genes were validated through single-cell sequencing, receiver operating characteristic analysis, and Muscle immunohistochemistry in a high-fat-diet (HFD) induced mouse model. Further analyses comprised immune infiltration profiling, pathway enrichment, and transcriptional regulation analysis. Additionally, we explored the relationships between key genes and autophagy, ferroptosis, and immunity responses. Finally, we predicted and evaluated potential therapeutic compounds via the CMap database and molecular docking.

**Results:**

SO incidence in China increased significantly from 16.1% (2011) to 20.4% (2018). Machine learning identified ALDH1A3, CSF1R, and PHGDH as key genes. These genes were validated in external muscle single-cell datasets, demonstrating robust diagnostic performance with AUC values exceeding 0.72 across four independent GEO cohorts. Following an HFD intervention in mice, ALDH1A3 and CSF1R expression in muscle tissue was significantly upregulated, while PHGDH showed a consistent upward trend that did not reach statistical significance. Immune infiltration analysis revealed a significant increase in resting NK cells in both obesity and sarcopenia states. Functional enrichment analyses using Gene Ontology and Kyoto Encyclopedia of Genes and Genomes linked the genes to transcriptional regulation pathways. The Cisbp_M4923 motif was identified as the most relevant transcription factor binding site. Finally, molecular docking simulations indicated stable binding of the top candidate compound, Birinapant, to the key gene targets.

**Conclusion:**

ALDH1A3, CSF1R, and PHGDH serve as potential co-morbid biomarkers for SO.

## Author summary

A lack of standardized protocols currently hinders the elucidation of interactive mechanisms and the development of treatments for sarcopenia-obesity comorbidity. To bridge this gap, we developed a comprehensive, multi-modal computational framework. Our workflow commenced with an 8-year longitudinal assessment of clinical burden using the CHARLS database. Subsequent integrated bioinformatics combining differential expression analysis, weighted gene co-expression network analysis, and cross-referencing with predicted gut microbiota metabolite targets identified ALDH1A3, CSF1R, and PHGDH as key comorbid genes. The key genes were rigorously validated across two external single-cell datasets, four independent GEO cohorts, and via immunohistochemistry in a high-fat-diet induced murine model. Upstream/downstream pathway and immune infiltration analyses further delineated their functional networks. Finally, leveraging the CMap database and molecular docking simulations, we screened and validated Birinapant as a promising therapeutic candidate. Collectively, this integrative framework establishes a systematic methodology for the mechanistic exploration and therapeutic targeting of complex comorbidities.

## Introduction

With economic development and changing lifestyles, the global spread of obesity has indeed become one of the most serious public health challenges. The prevalence of severe obesity is projected to double from 10% to 20% between 2020 and 2035, highlighting that the global prevalence of obesity remains at unacceptably high levels [1]. Obesity is a multifactorial, chronic, relapsing, non-communicable disease characterized by an abnormal and/or excessive accumulation of body fat [2]. Obesity management is indeed a critical topic that must be emphasized in future public health strategies.

In addition to the globalization of obesity, the contemporary reduced physical activity has contributed to a progressive increase in sarcopenia incidence [3]. Sarcopenia is defined as an age-related loss of muscle mass, strength, or physical performance [4]. It is estimated that the prevalence of sarcopenia ranges from 10% to 16% in the elderly population globally, with variations depending on diagnostic criteria and population demographics [5]. Sarcopenia is significantly associated with numerous adverse health outcomes, such as reduced overall and progression-free survival, falls, fractures, metabolic disorders, and increased mortality in the general population [6].

The strong correlation between obesity and sarcopenia has prompted the formulation of sarcopenic obesity (SO), which was established in 2022. In 2022, the European Society for Clinical Nutrition and Metabolism (ESPEN) and the European Association for the Study of Obesity (EASO) reached an expert consensus on the definition of SO [7], which is defined as obesity accompanied by low skeletal muscle mass, strength and/or function [8]. Therefore, this study first systematically examined the changing incidence trends of SO in the Chinese population using longitudinal follow-up data from the China Health and Retirement Longitudinal Study (CHARLS) from 2011 to 2018, preliminarily revealing the significant implications of SO for epidemiological management. However, due to the pathogenesis of SO remaining incompletely understood, effective and specific pharmacological interventions are currently lacking [9]. The gut microbiota is reshaping therapeutic paradigms for comorbid diseases by mediating cross-organ communication through regulatory networks like the gut-brain [10], gut-liver [11], and gut-muscle axes [12]. Therefore, elucidating the mechanisms by which the gut microbiota influences comorbidity offers a promising avenue for developing synergistic therapeutic strategies. To this end, this study integrated four machine learning methods (LASSO, XGBoost, SVM-RFE, and Random Forest) with gut microbiota metabolite target information to perform preliminary screening of transcriptome data from the Gene Expression Omnibus (GEO) database, thereby identifying key genes associated with obesity and sarcopenia comorbidity. We next validated the cellular expression patterns and diagnostic utility of key genes using two independent single-cell RNA sequencing (scRNA-seq) datasets and three independent transcriptomic datasets from GEO. Furthermore, we employed a high-fat diet (HFD)-induced mouse model to confirm the spatial distribution of key genes at the tissue level using immunohistochemistry (IHC). Following the definition of key genes, this study systematically analyzed the upstream and downstream pathways of these genes through a comprehensive approach. This approach integrated immune cell infiltration analysis, GeneMANIA-based functional association (GMFA), Gene Ontology (GO) and Kyoto Encyclopedia of Genes and Genomes (KEGG) enrichment analysis, regulatory network construction, and miRNA analysis. Additionally, the study examined the association between key genes and the immune, autophagy, and ferroptosis processes, which are of particular interest to researchers. Finally, to propose potential therapeutic strategies, we used the Connectivity Map (CMap) database to predict candidate compounds targeting these key genes. The interaction patterns were then visualized and validated through molecular docking simulations. In summary, this study established a comprehensive research framework for obesity and sarcopenia through epidemiological trend analysis, identification and multidimensional validation of key genes, systematic exploration of upstream and downstream pathways, and prediction of potential therapeutic agents. As SO remains a critical public health challenge, this bioinformatics investigation into its shared genetic mechanisms provides novel insights that may help elucidate its underlying pathology and inform future therapeutic strategies.

## Materials and methods

### Ethics statement

All animal experiments were approved by the Animal Ethics Committee of the First Affiliated Hospital, Zhejiang University School of Medicine, and were conducted in compliance with relevant guidelines and regulations.

### CHARLS

This study retrospectively analyzed data from the CHARLS waves of 2011, 2015, and 2018. Participants with missing data for key anthropometric measures, like height, weight, waist circumference, grip strength, and walking speed, were excluded, yielding final samples of 5,742, 6,379, and 8,064 participants for the 2011, 2015, and 2018 waves, respectively. Obesity was defined as meeting at least one of the following criteria: a body mass index ≥ 28 kg/m², a waist circumference ≥ 85 cm for men, or ≥ 80 cm for women. Sarcopenia was defined by the presence of any one of the following: a walking speed ≤ 1 m/s, a grip strength < 28 kg in men, or < 18 kg in women. Participants who met the criteria for both obesity and sarcopenia were identified as having SO.

### Data download

The obese GSE94752 and the sarcopenic GSE1428 datasets from the NCBI GEO public database [13] were downloaded for differentially expressed genes (DEGs) identification. Furthermore, the obese GSE163830 and the sarcopenic GSE172410 datasets were downloaded for single-cell sequencing analysis. The datasets GSE69039 and GSE44000, associated with obesity, as well as GSE136344 and GSE8479, related to sarcopenia, were downloaded for the receiver operating characteristic (ROC) curve analysis.

### Identification of DEGs

The “limma (3.61.12)” R package [14] was used to analyze DEGs between the healthy and diseased cohorts. Genes with P-value  <  0.05 and |logFC| > 0.585 were classified as DEGs. To facilitate the visualization of these DEGs, volcano plots and heatmaps were generated utilizing the “pheatmap (1.0.12)” and “ggplot2 (3.5.2)” R packages. Additionally, Venn diagram software was utilized to identify the DEGs.

### Weighted gene co-expression network analysis (WGCNA)

The co-expression network was constructed from the dataset using the “WGCNA (1.73)” R package [15]. The weighted adjacent matrix was converted into a Topological Overlap Matrix (TOM). A hierarchical clustering tree was then constructed based on the TOM. The resulting clustering tree delineates gene modules, with each branch and its corresponding color representing a distinct module. This method groups genes with highly correlated expression into the same module, effectively categorizing them by shared functional profiles.

### Identification of targets of gut microbiota metabolites

An initial set of gut microbiota metabolites and their putative human targets was sourced from the gutMGene v2.0 database [16]. The canonical SMILES (Simplified Molecular Input Line Entry System) notations for these metabolites were retrieved from the PubChem database. Targets for gut microbiota metabolites were obtained from the SEA database [17].

### Machine learning

To identify key genes, we applied four machine learning models. The LASSO model was constructed using the “glmnet (4.1.8)” package, the XGBoost model using the “XGBoost (1.7.8.1)” package, the SVM-RFE model using the “e1071 (1.7.16)” package, and the random forest model using the “randomForest (4.7.1.2)” package.

### Single‑cell sequencing analysis

The Single‑cell data were analyzed with the “Seurat (5.1.0)” R package [18], and then tSNE analysis was conducted to identify the spatial links between each pair of clusters. The clusters were then annotated using the “celldex (1.16.0)” R package.

### ROC curve analysis

To evaluate the diagnostic efficacy of ALDH1A3/CSF1R/PHGDH in obesity and sarcopenia, we performed the ROC curve analysis using the “pROC (1.18.5)” R package. The area under the ROC curve (AUC) was calculated to quantify the overall diagnostic performance of each gene.

### Animals

Six-week-old male C57BL/6J mice were obtained from GemPharmatech Co., Ltd. (Jiangsu, China). Mice were housed under a 12-hour light-dark cycle in a temperature-controlled environment with ad libitum access to water and a standard chow diet for 12 weeks. A second cohort of mice received an HFD (D12492, Research Diets, New Brunswick, NJ, USA) for 12 weeks. Euthanasia was performed by intraperitoneal injection of a ketamine (100 mg/kg) and enflurane (10 mg/kg) cocktail, followed by cervical dislocation. Following euthanasia, skeletal muscle and white adipose tissue were harvested for subsequent analysis.

### Small animal body composition analyzer

Body composition was measured using a low-field nuclear magnetic resonance analyzer (Suzhou Nuimag Analytical Instrument Corporation) for awake small animals, which quantified the mass of fat, lean tissue, and free fluid. The proportions of fat, lean tissue, and free fluid mass relative to total body weight were then calculated.

### IHC

Following dewaxing, antigen retrieval was performed using a citrate buffer (pH 6.0). Endogenous peroxidase activity was quenched by incubation with 3% hydrogen peroxide for 15 minutes. Sections were then blocked with 10% normal goat serum for 30 minutes at room temperature and subsequently incubated overnight at 4°C with the following primary antibodies: anti-ALDH1A3 (Abcam, USA, Cat. No. ab129815, 5 µg/ml), anti-CSF1R (Abcam, USA, Cat. No. ab254357, 1:100), and anti-PHGDH (ProteinTech, Wuhan, Cat. No. 65791, 1:4000). After washing, a species-matched secondary antibody was applied for 60 minutes at room temperature, followed by development with 3,3’-diaminobenzidine. Stained sections were imaged using an Olympus BX53 microscope, and the combined positive fraction was quantified using ImageJ software.

### Assessment of immune infiltration patterns

The CIBERSORT v1.03 algorithm quantified 22 immune cell types of both diseased and healthy samples. Significant differences were tested using Student’s t-tests and visualized using the “ggpubr (0.6.0)” R package. The connection between ALDH1A3/CSF1R/PHGDH and the proportions of immune cells was explored with the “corrplot (0.95)” R package, with the results displayed in a lollipop chart created with the “ggplot2 (3.5.2)” R package.

### GMFA network analysis

We employed GMFA network analysis [19] to identify 10 additional genes for ALDH1A3/CSF1R/PHGDH, prioritized based on their strongest associations within the gene-gene network. GMFA integrates three key parameters—co-expression, genetic interactions, and physical interactions—which significantly enhance the precision of therapeutic target identification.

### GO and KEGG analysis

The “Functional Annotation” feature of the Database for Annotation, Visualization, and Integrated Discovery (DAVID) [20] was employed to perform GO and KEGG enrichment analyses related to DEGs and additional genes for ALDH1A3/CSF1R/PHGDH after GMFA network analysis.

### Regulatory network analysis

The R package “RcisTarget (1.25.0)” [21] was used in this study to predict transcription factors. The normalized enrichment score (NES) of a motif is determined by the total number of motifs in the database. Besides the motifs identified in the source data, we deduced additional annotations based on motif similarity and gene sequence analysis. To ascertain the overexpression of each motif, the AUC for each motif-gene set pair was first determined based on the recovery curve calculation of the gene set in relation to motif ordering. The AUC values of all motifs in the gene set were used to calculate the NES of each motif. We utilized rcitarget.hg19.motifdb.cisbpont.500 bp as the gene-motif rankings database.

### miRNA analysis

MicroRNAs (miRNAs) are established regulators of gene expression. To investigate whether specific miRNAs participate in regulating the transcription or degradation of hub genes, we conducted miRNA analysis. Specifically, we identified miRNAs associated with hub genes using the miRcode database [22] and visualized the miRNA network utilizing Cytoscape (3.9.1) software.

### Relationship between ALDH1A3/CSF1R/PHGDH and key regulatory mechanisms

Genes associated with immunity, autophagy, and ferroptosis were sourced from the GeneCards database (https://www.genecards.org/). For the top 20 genes from each category, we extracted their expression levels and analyzed their correlation with ALDH1A3, CSF1R, and PHGDH expression in both obesity and sarcopenia cohorts. These correlations are visualized in a bubble chart.

### Potential therapeutic drugs predication

A promising tool for drug screening is the CMap (https://clue.io/) database, which can forecast molecularly targeted drugs based on DEGs [23]. In this study, we predicted potential chemical drugs for the treatment of obesity and sarcopenia using gene expression profiling and the CMap database.

### Molecular docking validation

Molecular docking was performed with the first core compounds from this study as ligands and the ALDH1A3/CSF1R/PHGDH gene product as the receptor. Initially, the 3D structures of the ligands were retrieved from the PubChem database (https://pubchem.ncbi.nlm.nih.gov/) [24], and the corresponding 3D structures of the targets were obtained from the RCSB protein database(https://www.rcsb.org/) [25]. Then, the molecular structures were optimized using Chimera (1.16), the docking box parameters were set using Autodock Vina (v1.2.3), the docking was performed using AutoDock KV1.2.3, and the results were visualized with Chimera 1.16 [26].

## Results

### Technology roadmap

Depicted in Fig 1.

**Fig 1:**
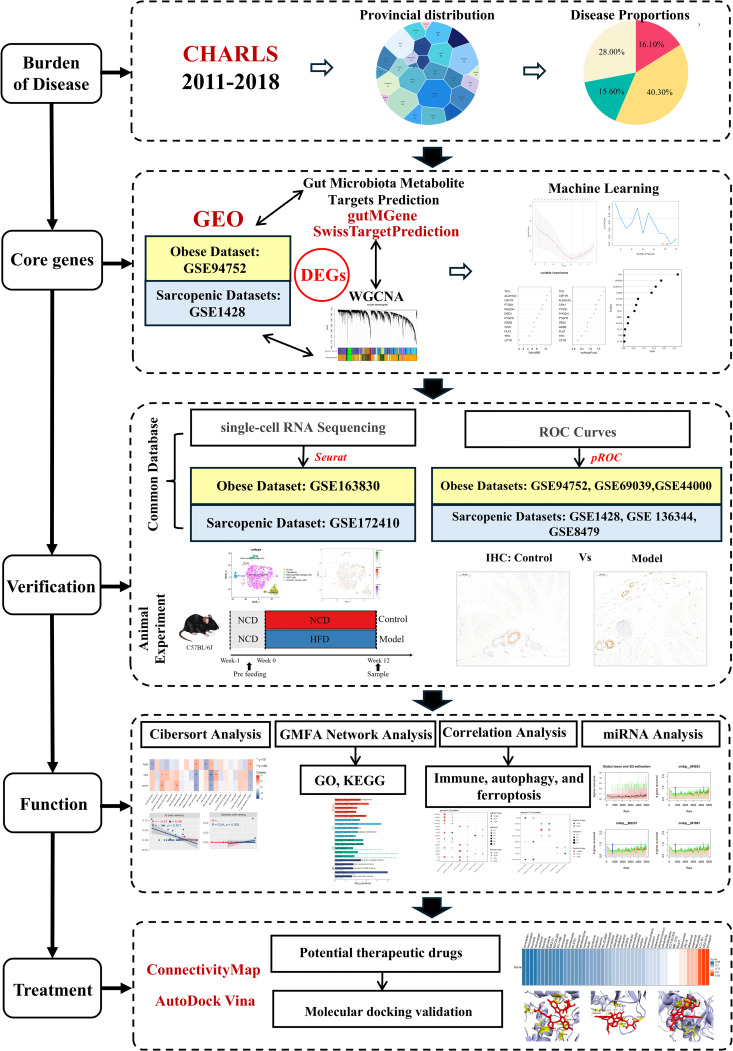
Flow chart.

### Clinical burden of SO

**Fig 2:**
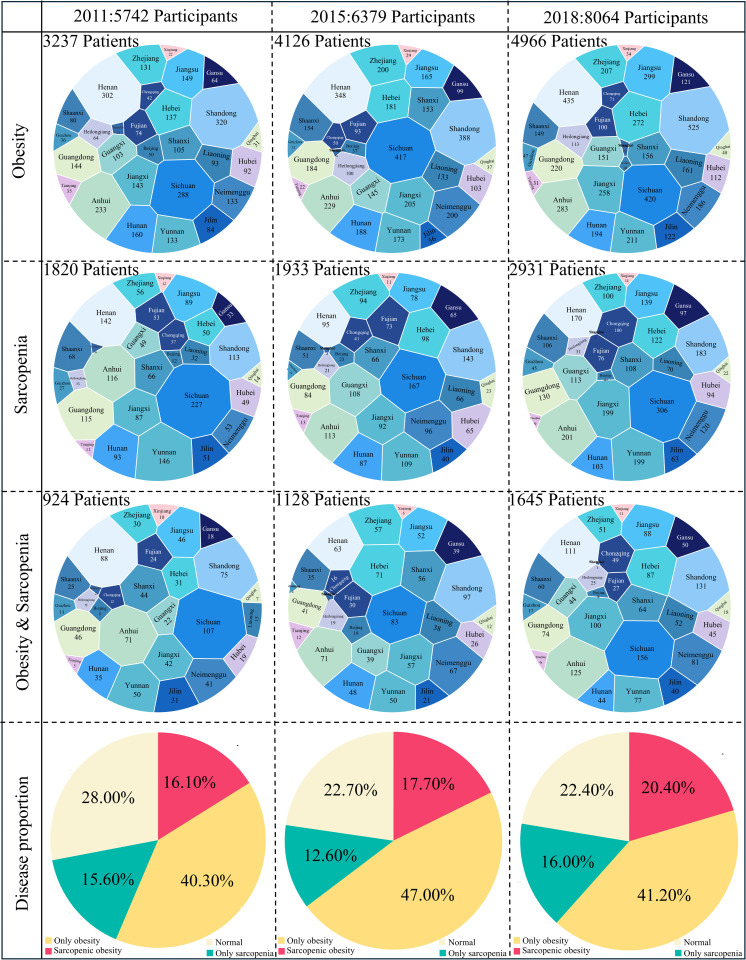
Clinical burden of SO. National prevalence, regional distribution and proportion of obesity, sarcopenia, and SO in China, 2011-2018.

In 2011, the prevalence of simple obesity was 40.30% (all obese: 3,237), while the prevalences of simple sarcopenia and SO were 15.60% (all sarcopenic: 1,820) and 16.10% (n = 924), respectively. By 2015, the prevalence of simple obesity had risen to 47.00% (all obese: 4,126). In contrast, the prevalence of simple sarcopenia was 12.60% (all sarcopenic: 1,933), and that of SO was 17.70% (n = 1,128). In 2018, the figures were 41.20% (all obese: 4,966) for simple obesity, 16.00% (all sarcopenic: 2,931) for simple sarcopenia, and 20.40% (n = 1,645) for SO. Over the study period, the prevalences of both sarcopenia and SO demonstrated an upward trend, while the prevalence of obesity remained persistently high (Fig 2). The steady increase in SO incidence highlights a long-term economic burden on healthcare systems.

### Screening of intersective DEGs

**Fig 3:**
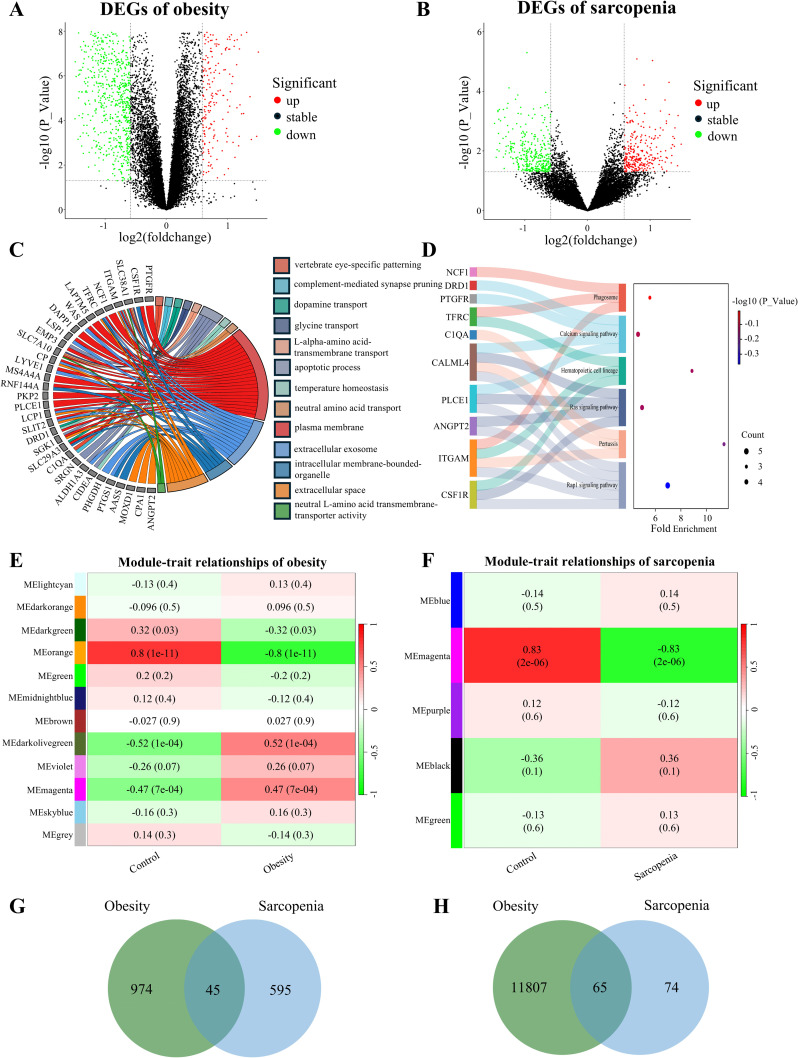
Identification of DEGs. **(A-B)** Volcano plot of DEGs associated with obesity and sarcopenia, where green represents down-regulated DEGs and red represents up-regulated DEGs. **(C-D)** GO and KEGG enrichment analysis of DEGs. **(E-F)** Heatmap of the correlation between module characteristic genes and obesity and sarcopenia; blue indicates negative correlation, and red indicates positive correlation. **(G)** Venn diagrams illustrate the co-upregulated and co-downregulated DEGs between obesity and sarcopenia. **(H)** Venn diagrams identify module intersection genes and hub genes.

Series Matrix Files for the obesity-related dataset GSE94752 and the sarcopenia-related dataset GSE1428 were downloaded from the NCBI GEO database. P value < 0.05 and |logFC| > 0.585 were the criteria. In the obese database, 1019 DEGs were identified, including 767 upregulated and 252 downregulated genes (Fig 3A). As for the sarcopenic databases, 640 DEGs were identified, including 353 upregulated and 287 downregulated genes (Fig 3B). We intersected the up- and down-regulated DEGs of the two diseases using Venn diagram software, yielding 45 intersecting DEGs (Fig 3G). Expression levels of the 45 DEGs across all samples are presented as heatmaps (S1A-B Fig). Functional enrichment analysis of the 45 DEGs revealed significant associations with specific GO and KEGG pathways, including plasma membrane and Rap1 signal pathway (Fig 3C-D).

### Coexpression network construction and hub module identification

WGCNA was performed to identify co-expression modules associated with obesity and sarcopenia. For the obese GSE94752 dataset, a soft-thresholding power of 4 was selected (S1C Fig). Gene modules were then identified using the TOM. A total of 12 gene modules were found, namely, lightcyan (2129), darkorange (284), darkgreen (2953), orange (11872), green (873), midnightblue (237), brown (1309), darkolivegreen (33), violet (37), magenta (472), skyblue (88), and grey (1). Among these, the orange module showed the strongest correlation with obesity (cor = -0.8, *p* = 1e-11) (Fig 3E). Subsequently, an analogous WGCNA was performed on the sarcopenic GSE1428 dataset, with a soft-thresholding power of 2 (S1E Fig). This analysis yielded 5 modules, namely, blue (13144), magenta (139), purple (129), black (79), green (104). The magenta module exhibited the strongest correlation with sarcopenia (cor = -0.83, *p* = 2e-06) (Fig 3F). Finally, the 11872 genes in the orange module and 139 genes in the magenta module were intersected, identifying 65 overlapping genes (Fig 3H).

### Identification of candidate genes

**Fig 4:**
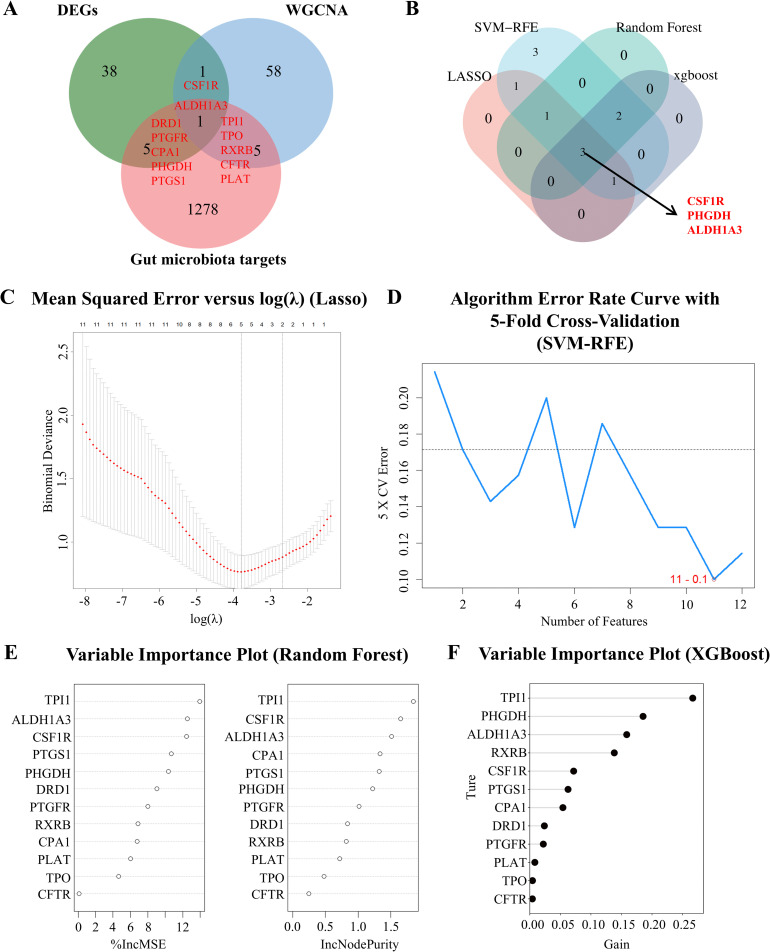
Identification of key genes. **(A)** Venn diagrams illustrate the potential key genes between DEGs, genes from the WGCNA model, and microbiota metabolite target genes. **(B)** Venn diagrams illustrate the key genes via four machine learning models. **(C)** Mean Squared Error versus log(λ) for the Lasso Model. **(D)** SVM-RFE Algorithm Error Rate Curve with 5-Fold Cross-Validation. **(E)** Variable Importance Plot for the Random Forest Model. **(F)** Variable Importance Plot for the XGBoost Model.

To investigate the role of gut microbiota in comorbid conditions, we retrieved all metabolites from the gutMGene v2.0 database and predicted 1289 potential targets of the metabolites using the SEA database. Intersection of these 1289 predicted targets with 45 DEGs and 65 module genes from WGCNA revealed 12 overlapping genes (Fig 4A). These 12 genes were considered candidate genes that met the criteria for transcriptomic comorbidity intersections, exhibit modular properties, and were potentially regulated by gut microbiota metabolites.

### Key genes identified by machine learning

To identify key genes from the candidate set, we employed four machine learning methods, including LASSO, SVM-RFE, XGBoost, and Random Forest. Three consensus key genes, namely ALDH1A3, CSF1R, and PHGDH, were identified across all four methods (Fig 4B-F). Specifically, LASSO regression selected six candidate genes: ALDH1A3, CSF1R, CPA1, PHGDH, RXRB, and CFTR. SVM-RFE selected 11 features: PHGDH, CPA1, TPI1, PTGFR, PLAT, TPO, CFTR, PTGS1, ALDH1A3, CSF1R, and RXRB. XGBoost ranked gene importance highlighted six genes: TPI1, ALDH1A3, CSF1R, PHGDH, PTGS1, and CPA1. Similarly, Random Forest analysis prioritized TPI1, PHGDH, ALDH1A3, RXRB, CSF1R, and PTGS1.

### External single-cell dataset validation

To evaluate the diagnostic potential of the three key genes for obesity and sarcopenia, we validated their performance in two single-cell datasets and four transcriptomic datasets.

**Fig 5:**
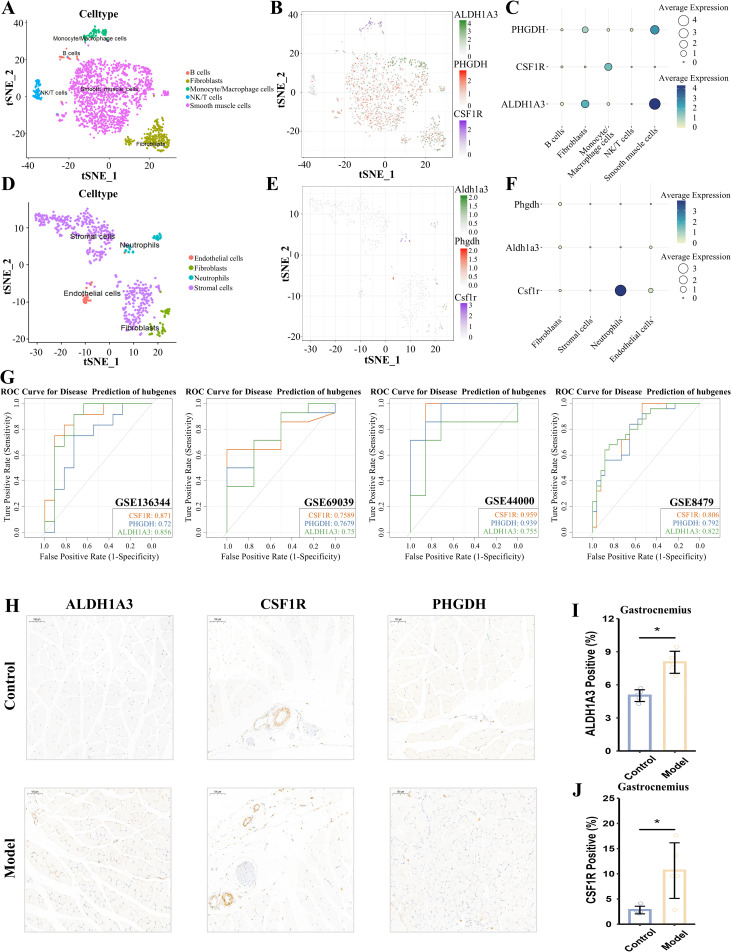
Verification of key genes. **(A)** Cellular subtypes of obesity. **(B)** Scatter plots of the expression of ALDH1A3, CSF1R, and PHGDH in GSE163830. **(C)** Average expression of ALDH1A3, CSF1R, and PHGDH in cellular subtypes in obesity. **(D)** Cellular subtypes of sarcopenia. **(E)** Scatter plots of the expression of ALDH1A3, CSF1R, and PHGDH in GSE172410. **(F)** Average expression of ALDH1A3, CSF1R, and PHGDH in Cellular subtypes in sarcopenia. **(G)** ROC curves of ALDH1A3, CSF1R, and PHGDH in GSE69039, GSE44000, GSE136344, GSE8479. **(H)** Representative immunohistochemical staining of the gastrocnemius muscle (scale bar, 100 μm). **(I-J)** Quantification of immunohistochemical staining intensity for **(I)** ALDH1A3, **(J)** CSF1R.

Cells in the GSE163830 dataset were annotated into five distinct cell types, including B cells, fibroblasts, monocyte/macrophage cells, NK/T cells, and smooth muscle cells (Fig 5A). Similarly, cells in the GSE172410 dataset were classified into four major types, including endothelial cells, fibroblasts, neutrophils, and stromal cells (Fig 5D). The expression patterns of ALDH1A3, CSF1R, and PHGDH across cell types in both datasets are visualized in Fig 5B, 5E, with quantification presented as bubble plots (Fig 5C, 5F). In GSE163830, PHGDH was expressed in all five cell types, while CSF1R and ALDH1A3 were absent only in NK/T cells. In GSE172410, CSF1R was significantly expressed in neutrophils.

### External transcriptome dataset validation

The three genes demonstrated strong diagnostic performance across four external datasets (Fig 5G). In the sarcopenia dataset GSE136344, ALDH1A3, CSF1R, and PHGDH achieved AUCs of 0.856, 0.871, and 0.720, respectively. In the obesity dataset GSE69039, their AUCs were 0.750, 0.759, and 0.768. In another obesity dataset GSE44000, the AUCs were 0.755, 0.959, and 0.939. Finally, in the sarcopenia dataset GSE8479, the AUCs were 0.822, 0.806, and 0.792, respectively. The AUCs for the original GSE94752 and GSE1428 datasets are presented in S2A and S2B Fig.

### Histological verification

To validate the expression differences predicted by bioinformatics analysis, we established an HFD-induced mouse model for vivo confirmation. Compared to the control group, HFD-induced mice showed a significant increase in body weight (29.14 ± 3.50 vs. 40.01 ± 2.42, *p* < 0.001) and a significant decrease in muscle mass (68.51 ± 3.15 vs. 48.75 ± 2.78, *p* < 0.001) (S2D and S2E Fig). Behavioral studies confirmed that this model exhibits both obesity and reduced muscle function, consistent with a comorbid phenotype. Representative IHC revealed significantly higher intensity for ALDH1A3 (5.02 ± 0.53 vs. 8.04 ± 1.00, *p* < 0.001) and CSF1R (2.81 ± 0.76 vs. 10.64 ± 5.52, *p* = 0.014) in the experimental group compared to controls. In contrast, PHGDH (2.22 ± 1.06 vs. 3.06 ± 1.44, *p* = 0.323) staining intensity did not differ significantly between groups (Fig 5H-J).

By integrating external single-cell and transcriptome datasets with immunohistochemical validation in mouse models, we confirmed at the cellular, transcriptional, and protein levels that the three key genes identified through bioinformatics analysis exhibit distinct expression patterns.

### Immune cell infiltration

**Fig 6:**
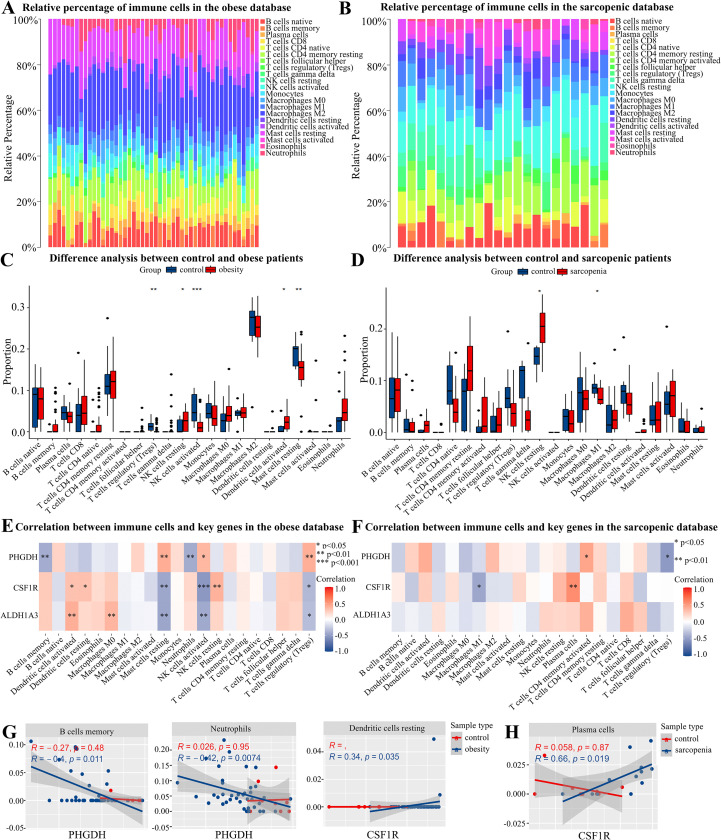
The immune infiltration cell analysis between control and disease samples. **(A)** Proportion of immune cell infiltration in control and obese samples. **(B)** Proportion of immune cell infiltration in control and sarcopenic samples. **(C)** Differences between control and obese samples for 22 immune cells infiltration. **(D)** Differences between control and sarcopenic samples for 22 immune cells infiltration. **(E)** Heatmap of correlation between the proportion of 22 immune cells and ALDH1A3, CSF1R, PHGDH in the obese dataset. **(F)** Heatmap of correlation between the proportion of 22 immune cells and ALDH1A3, CSF1R, PHGDH in the sarcopenic dataset. **(G)** The expressions of CSF1R and PHGDH were significantly associated with specific immune cell populations in obese cohorts. **(H)** The expression of CSF1R was significantly associated with plasma cell populations in sarcopenic cohorts.

Given the presence of immune cells in the single-cell dataset and the role of CSF1R as a macrophage surface marker, we conducted immune cell infiltration analysis to assess their contribution to the comorbid phenotype. The proportions of immune cell infiltration in control and disease samples are shown in Fig 6A, 6B.

In the obesity group, the proportions of activated dendritic cells (DCs) (*p* < 0.05) and resting natural killer cells (NKs) (*p* < 0.05) were significantly elevated compared with the control group, while the proportion of regulatory T cells (Tregs) (*p* < 0.01), activated NKs (*p* < 0.001), and resting mast cells (*p* < 0.01) was markedly reduced (Fig 6C). In the sarcopenia group, the proportion of resting NKs (*p* < 0.05) was significantly increased, while M1 macrophages (*p* < 0.05) were significantly decreased (Fig 6D).

Correlations between the three key genes and 22 immune cell types across all samples are presented as heatmaps in Fig 6E, 6F. In obese samples, PHGDH was negatively correlated with memory B cells (R = -0.4, *p* = 0.011) and neutrophils (R = -0.42, *p* = 0.0074). Conversely, CSF1R was positively correlated with resting DCs (R = 0.34, *p* = 0.035) (Fig 6G). In sarcopenic samples, CSF1R expression was positively correlated with plasma cells (R = 0.66, *p* = 0.019) (Fig 6H). These findings indicate that immune cell profiles are significantly altered during the development of obesity and sarcopenia, and CSF1R and PHGDH may play regulatory roles in this process.

### GMFA network analysis

**Fig 7:**
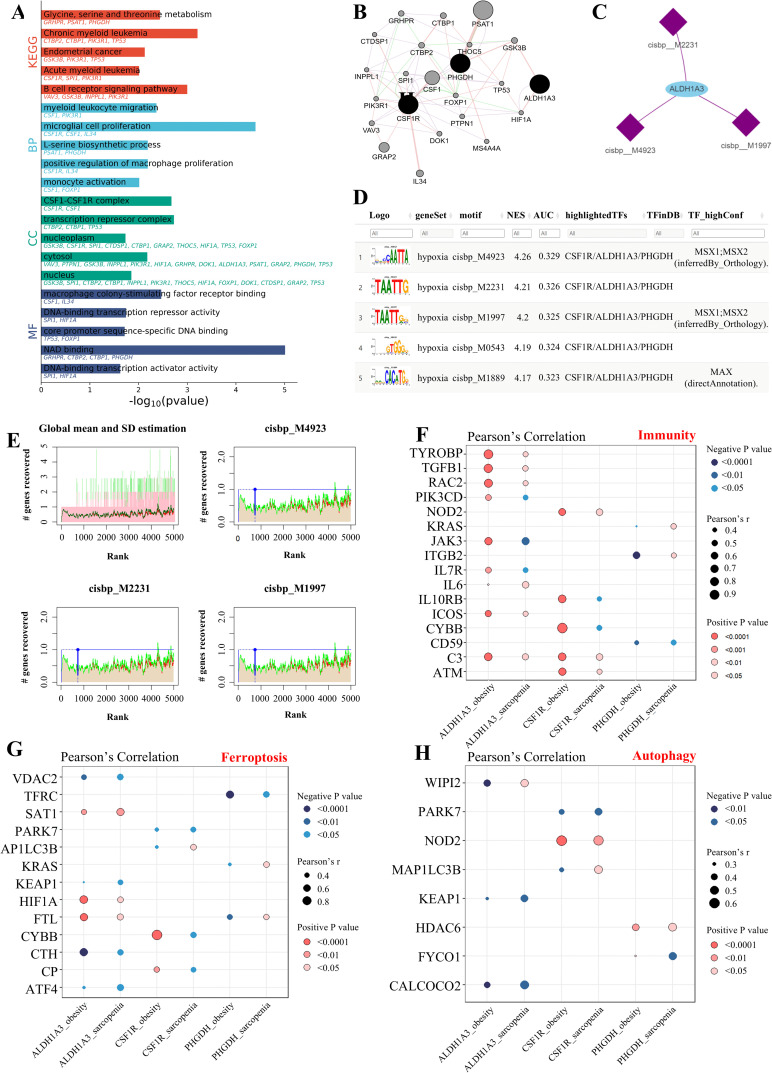
Functional and pathway enrichment analysis. **(A)** GO and KEGG enrichment analysis of gene set from ALDH1A3, CSF1R, and PHGDH via GMFA. **(B)** Functionally related genes of ALDH1A3, CSF1R, and PHGDH. **(C)** MiRNA networks associated with ALDH1A3, CSF1R, and PHGDH, where blue denotes mRNA and purple denotes miRNA. **(D)** Display of the highest motif enrichment based on AUC, incorporating NES, AUC, and TF_highConf. **(E)** The three motifs with the highest AUC values are presented. The red line represents the average recovery curve of each motif, the green line represents the mean plus standard deviation, and the blue line represents the recovery curve of the current motif. The maximum enrichment level is selected at the point where the distance (mean + standard deviation) between the current motif and the green curve is maximized. **(F-H)** Correlation of ALDH1A3, CSF1R, and PHGDH with immune, ferroptosis, autophagy factors.

To explore functional associations among key genes and predict their potential synergistic mechanisms, we conducted a GMFA network analysis. This analysis identified 20 additional genes associated with the three key genes, including CSF1 and GRAP2, among others (Fig 7B). This gene network was then subjected to functional enrichment analysis using GO and KEGG databases (Fig 7A). Notably, the significant BP terms included microglial cell proliferation; the key CC terms comprised transcription repressor complex; the key MF terms comprised NAD binding; the key KEGG terms included chronic myeloid leukemia.

### Transcription factor binding motif analysis

As for key transcriptional CC terms, we then examined the relationship between the key genes and transcription factors. The cumulative recovery curve (Fig 7E), motif-TF annotation, and the selection analysis results of significant genes were subjected to enrichment analysis for these transcription factors. The hub gene-enriched motifs, including cisbp__M2231, cisbp__M4923, and cisbp__M1997, are shown in Fig 7C. The results revealed that the motif with the highest NES (4.26) was cisbp__M4923 (Fig 7D).

### Correlation with key regulatory mechanisms

We then assessed the expression correlations between the three key genes and the top 20 genes involved in immunity, autophagy, and ferroptosis pathways in the context of obesity and sarcopenia (Fig 7F–7H). The results showed that three key genes were strongly correlated with genes from all three pathways, suggesting potential regulatory roles in these processes.

### Potential drug prediction and molecular docking

**Fig 8:**
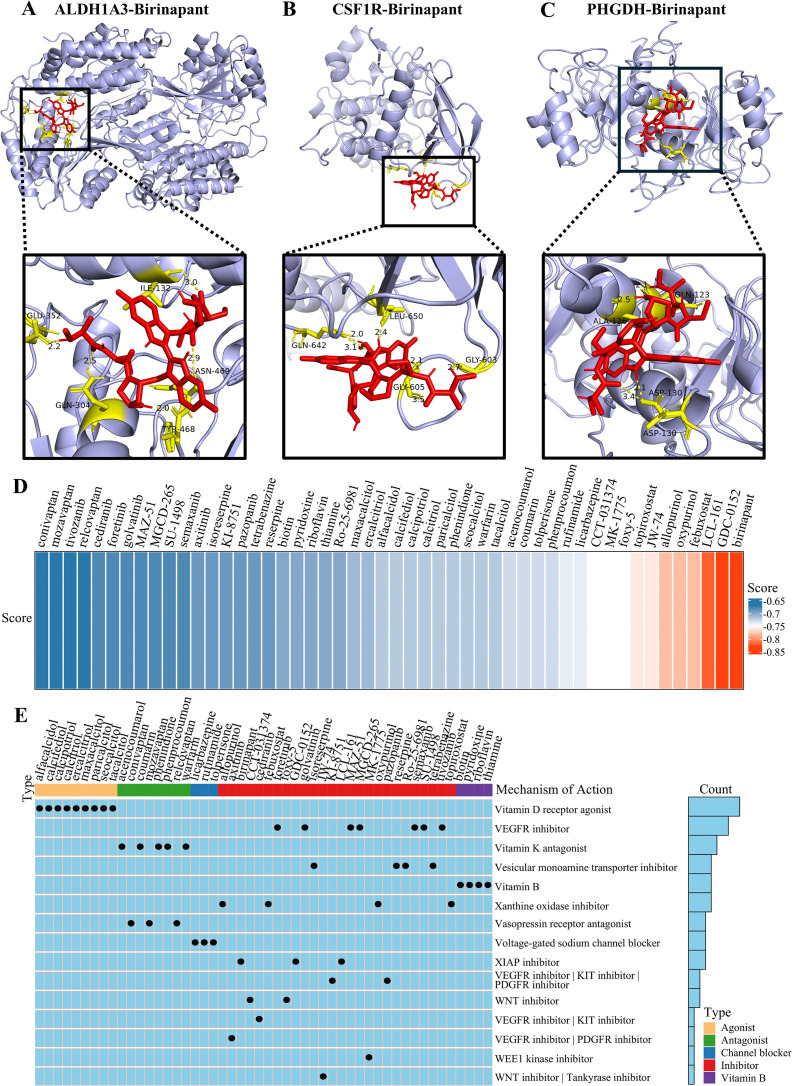
Prediction of potential therapeutic agents for obesity and sarcopenia. **(A-C)** Molecular Docking of Birinapant with Target Proteins ALDH1A3, CSF1R, and PHGDH. **(D)** Heatmap displaying the scores of potential therapeutic agents as predicted by the Connectivity Map database. **(E)** Schematic diagram of the mechanism of potential therapeutic drugs.

Following our analysis of the disease burden and comorbidity mechanisms of SO, we predicted potential therapeutic agents, providing a theoretical basis for novel drug development. We downloaded drug data from CMap and predicted potential therapeutic drugs based on the 12 potential key genes in Fig 4A. Among the candidate drugs, birinapant received the highest score and was identified as the most promising candidate for alleviating the comorbid condition (Fig 8D). The results of molecular docking of Birinapant with ALDH1A3, CSF1R, and PHGDH are presented in Fig 8A-8C. Birinapant has stable binding to all three key genes. The mode of action of the potential drug is presented in Fig 8E.

## Discussion

Obesity is closely associated with sarcopenia, a condition characterized by the interaction between adipose tissue and skeletal muscle function [27], where lipids and their derivatives are deposited intracellularly and intercellularly, leading to impaired energy uptake, and where cytokines secreted by the muscle exacerbate the development of chronic inflammation and hyperlipidemia [28]. This vicious cycle promotes the development of SO and leads to a worse prognosis than the disease alone. Research indicates that women afflicted with both sarcopenia and SO exhibit an elevated risk of mortality compared to those without these single conditions [29]. As the population ages and lifestyles evolve, the prevalence and global public health burden of sarcopenia will further increase. Therefore, investigating the mechanisms underlying the comorbidity between obesity and sarcopenia is clinically important for early identification and intervention of SO.

Analysis of the CHARLS database (2011–2018), comprising approximately 20,000 individuals, revealed an increasing trend in the incidence of SO in China. This trend indicates that SO already affects a substantial population and is poised to remain a condition with significant public health implications. Against this research backdrop, we integrated datasets on obesity and sarcopenia, applying differential gene expression analysis, WGCNA, and gut microbiota metabolite targets. Four distinct machine learning algorithms converged on three key genes: ALDH1A3, CSF1R, and PHGDH. The representativeness of these genes was validated by rigorously evaluating their expression patterns across two single-cell datasets and assessing their diagnostic value via ROC curve analysis in four independent GEO datasets. Furthermore, in an HFD-induced SO mouse model, the expression of these key genes was visually confirmed by IHC of muscle tissue. The genes identified through this multi-faceted approach demonstrated robust representativeness and stability. Notably, these three genes appear to contribute to SO pathogenesis through distinct yet interrelated pathways. Their specific pathogenic mechanisms are discussed in detail in the subsequent sections.

ALDH1A3 was identified as a key gene, located at the intersection of DEGs, key WGCNA modules, and the gut microbiota metabolite target cluster. Physiologically, ALDH1A3 is a member of the aldehyde dehydrogenase family that catalyzes the oxidation of all-trans retinal to retinoic acid, thereby regulating intracellular retinoic acid signaling [30]. In the context of obesity, increased pancreatic ALDH1A3 expression is a marker of β-cell dedifferentiation [31], a key pathological feature and a potential therapeutic target. In sarcopenia, however, the role of ALDH1A3 is less characterized. Nevertheless, it has been shown to modulate skeletal muscle satellite cell myogenic differentiation by regulating the cell cycle via retinoic acid formation [32], a mechanism that may influence sarcopenic pathology. Collectively, this evidence nominates ALDH1A3 as a promising therapeutic target for SO, given its implicated roles in both obesity-related metabolic dysfunction and sarcopenia-related impairment of muscle regeneration. Although PHGDH belongs to an enzyme family like ALDH1A3, it was not a member of the key WGCNA modules. PHGDH is the first and rate-limiting enzyme of the serine synthesis pathway [33]. This reaction initiates the serine synthesis pathway and concurrently generates NADH. In obesity models, PHGDH inhibition appears beneficial; myeloid PHGDH deficiency reverses diet-induced obesity [34], and adipocyte-specific PHGDH knockout improves glucose intolerance [35]. Conversely, in the context of sarcopenia, PHGDH overexpression promotes myocyte proliferation [36]. Therefore, the precise contribution of PHGDH to SO pathogenesis, given these opposing tissue-specific effects, remains to be elucidated. We therefore hypothesize that a dual-tissue targeting strategy, inhibiting PHGDH in adipocytes while promoting its activity in muscle, may represent a brave therapeutic approach for SO. Notably, the anti-obesity effect of PHGDH deficiency is partially mediated through the regulation of mitochondrial biogenesis in macrophages. This macrophage-centric mechanism provides a logical connection to CSF1R, which is also critically involved in macrophage biology. Among the key genes identified, CSF1R was the only one not linked to gut microbiota metabolite targets. As a receptor for the ligands CSF1 and IL-34, CSF1R is a master regulator of macrophage survival, proliferation, differentiation, and function [37]. In obesity, CSF1R inhibition has been shown to prevent diet-induced obesity and insulin resistance, thereby improving metabolic parameters [38,39]. In sarcopenia, CSF1R has been proposed as a key regulator of SO [40], although its potential as a therapeutic target remains unexplored. Currently, the associations between these key genes and SO are largely inferred from their established biological functions and indirect evidence. Therefore, elucidating their precise pathophysiological roles and underlying molecular mechanisms represents a critical direction for future research.

As noted in the introduction, the gut microbiota manages complex pathological conditions, such as comorbidities, may more effectively than single-agent therapies via microecological regulation. Consequently, we incorporated targets of gut microbiota metabolites into our selection of potential key genes. Both ALDH1A3 and PHGDH are targets of gut microbiota metabolites, which prompted an investigation into their specific interactions with the microbiota. Cui et al. demonstrated that trans-3-indolepropionic acid, a tryptophan metabolite from Peptostreptococcus anaerobius, transcriptionally upregulates ALDH1A3. Using retinaldehyde as a substrate, ALDH1A3 produces NADH, thereby inhibiting ferroptosis and promoting colorectal cancer [41]. Since lipid peroxidation induced by obesity exacerbates ferroptosis [42], dysregulation of ferroptosis-related factors further triggers ferroptosis in M2 macrophages and Treg cells, thereby disrupting the local immune microenvironment [43]. Ferroptosis contributes to sarcopenia by driving iron overload and reactive ROS accumulation within muscle tissue, which promotes oxidative damage and impairs regenerative capacity [44]. Given the key pathogenic role of ferroptosis in obesity and sarcopenia, upregulating ALDH1A3 could potentially ameliorate sarcopenia by inhibiting ferroptosis [45] and, via muscle enhancement, alleviate obesity. However, other evidence indicates that ferroptosis is reduced in adipocytes and that its promotion can mitigate obesity. Given this apparent paradox, we further investigated the relationships between the three key genes and the top 20 ferroptosis-related genes in our functional analyses. Similarly, the PHGDH-mediated serine pathway is a crucial component of gut microbiota metabolism and participates in hepatic lipid metabolism [46]. However, its direct link to sarcopenia remains poorly characterized. Nevertheless, both the serine metabolic pathway and the gut microbiota itself represent modifiable factors in the pathophysiology of sarcopenia. Although CSF1R is not a direct target of gut microbiota metabolites, the interaction between the gut microbiota and macrophages is critical for intestinal homeostasis and is a current research focus. Therefore, the modulation of the immune microenvironment by the gut microbiota represents a potential therapeutic pathway for ameliorating comorbidities.

As the preceding discussion illustrates, intricate functional connections exist among the three key genes. To further investigate their functions, we performed a series of downstream analyses, including GMFA, GO and KEGG pathway enrichment, and transcription analysis. The GO and KEGG analyses revealed significant enrichment of serine-related pathways, such as glycine, serine, and threonine metabolism and the L-serine biosynthetic process. This finding aligns with our earlier discussion and is likely primarily driven by PHGDH. Pathways related to NAD-binding and the transcription repressor complex were also notably enriched. Furthermore, transcriptional analysis identified cisbp_M4923 as the most relevant transcription factor. Given the established association of ALDH1A3 with ferroptosis, we investigated the relationships of three key genes with three key pathways, including autophagy, ferroptosis, and immunity, to elucidate their potential mechanisms in SO. The results indicate that ALDH1A3 exhibits largely consistent expression trends in both obesity and sarcopenia, whereas CSF1R demonstrates more opposing trends between the two conditions. In contrast, PHGDH showed low correlation with genes across all three key pathways. ALDH1A3 was predominantly positively correlated with immune-related genes in both conditions, a finding potentially explained by the essential role of ALDH1A3-regulated retinoic acid in adult immune function [47]. However, the correlation trends between ALDH1A3 and ferroptosis-related genes were inconsistent, underscoring the need for further validation of the underlying molecular mechanisms. Overall, the expression levels of the three key genes showed varying correlations with factors across the autophagy, ferroptosis, and immune pathways, suggesting complex interplay among these processes in SO.

Metabolic disorders and complications affecting organ tissues in individuals with obesity are linked to chronic inflammation within adipose tissue [48]. This inflammatory response is also observed in skeletal muscle, primarily characterized by heightened infiltration of immune cells and pro-inflammatory activation of interstitial cells and perimysial adipose tissue, which may exacerbate the condition of sarcopenia [49,50]. Consequently, research focusing on the immune microenvironment associated with obesity and sarcopenia is of paramount importance. Furthermore, both CSF1R and PHGDH are involved in immunoregulatory pathways, and all three key genes are significantly correlated with immune-related gene sets. These observations prompted an immune infiltration analysis. The analysis revealed that resting NKs were the only immune subset elevated in both obesity and sarcopenia. Furthermore, CSF1R expression was positively correlated with resting NKs infiltration specifically in obesity. Prior research has demonstrated that the selective ablation of CSF1R-positive NKs populations can mitigate obesity and insulin resistance [39]. Furthermore, this association between resting NKs and obesity has been reported in multiple bioinformatics studies [51,52]. The accumulation of resting NKs in obese individuals may reflect a dysregulation of activation. As aerobic glycolysis is a metabolic hallmark of NKs activation [53], the high glycolytic dependence of adipocytes may competitively deprive essential substrates for NKs [54], thereby inhibiting NKs activation. However, this proposed mechanism of metabolic competition requires further experimental validation. Recently, another study similarly found that resting NK cells are closely associated with sarcopenia [55]. Similarly, their increased activity post-exercise suggests a potential therapeutic avenue for its amelioration [56–58]. IL-15 is highly expressed in skeletal muscle, where it functions to inhibit fat deposition and promote skeletal muscle anabolic metabolism. IL-15 is also essential for the development, maintenance, and survival of NKs [59]. Within the vicious cycle of obesity and sarcopenia, IL-15 levels are diminished, potentially serving as a latent trigger for NKs immune senescence. As a crucial component of the innate immune system, the precise mechanisms by which NK cells contribute to SO pathogenesis, however, require further extensive experimental elucidation.

Prior research indicates that patients with SO face a poorer prognosis compared to those with single diseases. However, the exploration of the pathogenesis and shared etiological factors between obesity and sarcopenia using bioinformatics has been limited, highlighting the clinical importance of investigating the dual-disease mechanism to enhance the prognosis of SO. In this study, we have identified hub genes in obesity and sarcopenia, and analyzed potential regulators and therapeutic agents, contributing to a deeper understanding of the molecular mechanisms underlying the co-occurrence of these conditions. However, our study acknowledges certain limitations. Firstly, our study identified only 3 key genes. Other genes showing suggestive associations, along with the potential alternative or compensatory pathways they may regulate, constitute promising targets for future investigation. Secondly, the detailed molecular mechanisms of how hub genes, miRNAs, and transcription factors affect these diseases remain unclear. Lastly, the functions of the hub genes and the efficacy of potential therapeutic agents in vivo need to be verified by further experimental and clinical studies.

## Conclusion

Analysis of the CHARLS database confirms a rising incidence of SO, underscoring its critical public health relevance. An integrated multi-stage screening strategy, including DEGs analysis, WGCNA, and intersection with predicted gut microbiota metabolite targets, was employed to identify 12 initial candidate genes. These candidates were refined by four independent machine learning methods (LASSO, XGBoost, SVM-RFE, and Random Forest), ultimately identifying ALDH1A3, CSF1R, and PHGDH as high-confidence hub genes implicated in obesity and sarcopenia. The stability and diagnostic potential of the key genes were validated using two independent scRNA-seq datasets and ROC curve analyses across four independent GEO cohorts. Most critically, this study provided direct in vivo evidence by confirming the expression and spatial distribution of these genes via IHC of gastrocnemius muscle tissue in a HFD-induced mouse model of SO. Further investigation revealed that these key genes are significantly correlated with diverse immune cell infiltrates in both conditions, illuminating their role in shaping the immune microenvironment. GMFA, along with GO and KEGG pathway analyses, linked ALDH1A3, CSF1R, and PHGDH to significantly enriched pathways, including transcriptional regulation, with the Cisbp__M4923 motif emerging as the most relevant transcription factor binding pattern. Moreover, these genes were implicated in autophagy, ferroptosis, and immune responses. Finally, leveraging the CMap database, we identified potential therapeutic compounds; the top candidate, Birinapant, was subjected to molecular docking, which confirmed its stable binding potential with the key genes.

In summary, this study established a comprehensive computational biology research framework encompassing clinical burden assessment, key gene identification, external dataset and experimental validation, upstream/downstream pathway enrichment analysis, and potential drug discovery. This work advances the mechanistic understanding of the sarcopenia-obesity comorbidity and provides a foundation for developing novel therapeutic strategies.

## Supporting information

S1 Fig Identification of DEGs. (A-B) Heatmap displaying DEGs associated with obesity and sarcopenia. (C, E) Scaleless index and mean connectivity of individual soft thresholds for obesity and sarcopenia. (D, F) Cluster Dendrogram of obesity and sarcopenia, with different colors representing different modules.
(TIF)

S2 Fig Verification of key genes. (A-B) ROC curves of ALDH1A3, CSF1R, and PHGDH in GSE94752 and GSE1428. (C) Animal experiment flowchart. (D) Body weight of control and HFD mice (n = 8 per group) (E) Ratio of muscle mass to body weight (n = 8 per group). (F) Quantification of immunohistochemical staining intensity for PHGDH.
(TIF)
